# No Evidence of XMRV or Related Retroviruses in a London HIV-1-Positive Patient Cohort

**DOI:** 10.1371/journal.pone.0018096

**Published:** 2011-03-23

**Authors:** Eleanor R. Gray, Jeremy A. Garson, Judith Breuer, Simon Edwards, Paul Kellam, Deenan Pillay, Greg J. Towers

**Affiliations:** 1 Department of Infection and Immunity, University College London, London, United Kingdom; 2 Mortimer Market Centre, Camden Primary Care Trust, London, United Kingdom; 3 Pathogen Genetics, Wellcome Trust Sanger Institute, Cambridge, United Kingdom; Beckman Research Institute of the City of Hope, United States of America

## Abstract

**Background:**

Several studies have implicated a recently discovered gammaretrovirus, XMRV (Xenotropic murine leukaemia virus-related virus), in chronic fatigue syndrome and prostate cancer, though whether as causative agent or opportunistic infection is unclear. It has also been suggested that the virus can be found circulating amongst the general population. The discovery has been controversial, with conflicting results from attempts to reproduce the original studies.

**Methodology/Principal Findings:**

We extracted peripheral blood DNA from a cohort of 540 HIV-1-positive patients (approximately 20% of whom have never been on anti-retroviral treatment) and determined the presence of XMRV and related viruses using TaqMan PCR. While we were able to amplify as few as 5 copies of positive control DNA, we did not find any positive samples in the patient cohort.

**Conclusions/Significance:**

In view of these negative findings in this highly susceptible group, we conclude that it is unlikely that XMRV or related viruses are circulating at a significant level, if at all, in HIV-1-positive patients in London or in the general population.

## Introduction

Xenotropic murine leukaemia viruses (X-MLVs) are a class of endogenous gamma retroviruses. Xenotropic murine leukaemia virus-related virus (XMRV) is an X-MLV that has been detected in human samples and as such, is potentially the first gammaretrovirus to infect humans. XMRV has been detected in samples from patients with diseases such as prostate cancer and chronic fatigue syndrome, and has also been detected in 1–7% of healthy controls tested in the same studies [1]–[6]. The association between XMRV and human disease is controversial with some studies detecting XMRV in up to 87% of patients whilst others have failed to detect XMRV infection either in patient cohorts or in the general population [1]–[17].

Here we set out to assess the prevalence of X-MLVs, including XMRV, in an HIV-1-positive patient cohort in London. HIV-1-positive patients were investigated because those who have been infected by a sexual route, by intravenous drug use, by perinatal infection or iatrogenically are likely to have been at greater risk than the general population of other viral infections spread by similar routes (e.g. HBV, HCV, HTLV) [18]–[22]. Although no definitive route of infection has so far been found for XMRV, all four known human retroviruses, HIV-1, HIV-2, HTLV-1 and HTLV-2, share the same routes of transmission, namely the transfer of blood or other body fluids. We therefore hypothesised that if XMRV or related viruses were circulating in London they would be likely to be detected in HIV patients. We found no evidence for X-MLV or XMRV infection in 540 DNA samples purified from peripheral blood leukocytes of HIV-1 infected individuals.

## Materials and Methods

### Ethics Statement

The University College London Research Ethics Committee has specifically exempted this study from review because it was an assay development, and waived the need for consent due to the fact the patient material used was fully anonymised.

### Collection and screening of HIV-1–positive samples

Samples were collected from consecutive patients attending Mortimer Market Centre HIV service over a period of two months and were anonymised before processing. Patients were 4.5∶1 male:female, age range 15–85 yrs, median age 42 yrs, <1% intravenous drug users, 15% were born outside the UK. 80% of patients were on highly active anti-retroviral therapy (HAART); 94% of those treated had a viral load <50 copies/ml. Approximately 8 ml blood was collected into a BD-vacutainer containing EDTA, and stored at 4°C until processing. Genomic DNA from the buffy coat fraction was extracted using the QIAamp DNA kit (Qiagen) and eluted in 60 µl.

### TaqMan Polymerase Chain Reaction (TaqMan PCR)

**Figure 1:**
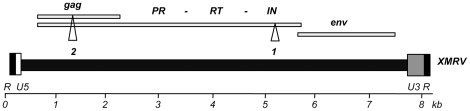
Primer and probe positions on the XMRV genome. Diagram of the XMRV genome with the sites of the primers marked.

TaqMan PCR of genomic DNA using primer sets 1 and 2 were performed as described (Figure 1, Table 1) [3], [12]. Sample levels were 5 µl per PCR reaction for controls, and ∼1000 ng DNA (usually 5 µl) for patient samples per PCR reaction. Positive controls for the TaqMan PCR were either a synthetic plasmid encoding the target XMRV-*int* sequence for primer set 1 or Balb/c mouse DNA (Sigma D4416) for primer set 2 (X-MLV-*gag*). Cycling conditions were 95°C for 15 secs and annealing/extension at 60°C for 1 min after an initial denaturation of 10 min. The HIV-1 positive samples were checked for PCR inhibitors by amplification of GAPDH as previously described [23].

**Table 1:** Sequences of TaqMan PCR primer/probe sets used to screen HIV-1-positive patient leukocyte DNA.

	Target	Reference	Primer and Probe sequences
1	XMRV *integrase*	[3]	F-CGAGAGGCAGCCATGAAGG
			R-GAGATCTGTTTCGGTGTAATGGAAA
			P-FAM-AGTTCTAGAAACCTCTACACTC-MGBNFQ
2	*X-MLV gag*	[12]	F-AACCGTTTGTCTCTCCTAAACCC
			R-GCAGGGTAAAGGGCAGATCG
			P-FAM-ACCGACAGCTCCCGTCCTCCCG-TAMRA

## Results

### No evidence for XMRV or X-MLV sequences in leukocyte DNA from a cohort of HIV-1 positive individuals

**Figure 2:**
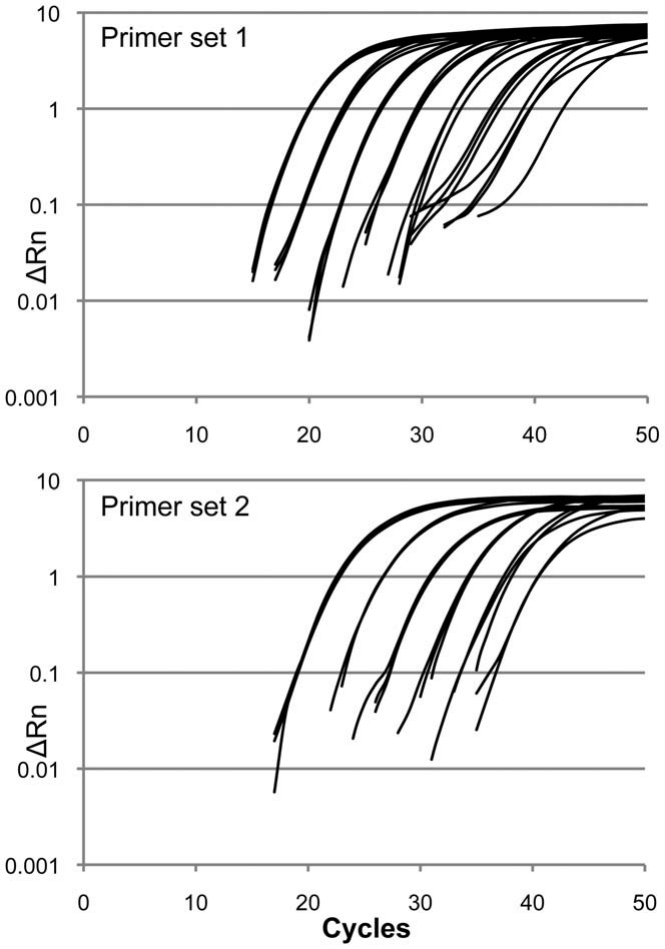
Detection of TaqMan positive controls used for HIV-1-positive leukocyte DNA sample screening. TaqMan PCR curves are shown for amplification of serial 10-fold dilutions of positive control DNA (A) Curves represent the triplicate amplification of dilutions of XMRV-*int*-encoding plasmid from 5×10^7^- 5 copies per PCR reaction using primer set 1. (B) Curves represent the triplicate amplification of dilutions of Balb/c genomic DNA from 200 ng–0.2 pg per PCR.

In order to assess the claim that XMRV infection is common in the human population we screened leukocyte DNA purified from anonymised blood samples of 540 HIV-1-positive patients visiting Mortimer Market HIV service. Approximately 20% of this cohort (∼108 patients) had not been treated with any anti-retroviral therapy. We performed TaqMan PCR using previously described primer sets 1 (XMRV-*int*) and 2 (X-MLV-*gag*) (Fig. 1, Table 1) [3], [12]. Primer set 1 detects XMRV but was designed to discriminate against related X-MLVs, whereas primer set 2 readily amplifies diverse X-MLV sequences present in the mouse genome [3], [12]. The positive control for primer set 1 was a plasmid containing the XMRV *integrase* target sequence and the positive control for primer set 2 was Balb/c mouse genomic DNA. We were able to detect (at the 50% probability level) as few as 5 copies of the XMRV-*int* plasmid and 0.2 pg (1/20^th^ of a genome) of Balb/c DNA respectively (Figs. 2A and 2B).

To assess the sensitivity of the PCRs when detecting XMRV integrated into genomic DNA, we extracted DNA from 22Rv1 cells. This cell line contains around 10 copies of the XMRV provirus [24]. Using primer sets 1 and 2, it was possible to reliably amplify 1 cell equivalent of DNA. To investigate the sensitivity of the assay through the entire extraction and amplification process, 0, 10, 50, 250 & 1000 22Rv1 cells were mixed with leukocytes taken from HIV-1-positive patients, blinded to the operator, then extracted using an identical protocol to the cohort samples and amplified using primer set 2 (Table 2). All cycle threshold (Ct) values were in the linear range of the assay (<40). Absolute values were not calculated, as we cannot be sure that XMRV/X-MLV sequences in the 22Rv1 cells exactly match the primer sequences. However, it was possible to detect ten 22Rv1 cells added to the buffy coat fraction extracted from 8 ml blood at Ct 35 (equivalent to approximately one 22Rv1 cell per 3 million white blood cells) (Table 2). No signal was obtained from the samples that did not have any 22Rv1 cells added.

**Table 2:** TaqMan PCR results from extraction and amplification control experiments.

Sample name	Number of 22Rv1 cells added to leukocyte sample	Expected number of XMRV genomes per PCR reaction (calculated from input)	Primer set 2 (X-MLV-*gag*) TaqMan PCR result (Ct)
A	10	8.3	35
B	250	208	31
C	0	0.00	No Ct
D	50	42	32
E	1000	833	28

22Rv1 cells were added to leukocyte samples taken from approximately 8 ml blood, extracted, and amplified using primer set 2. The expected number of copies of XMRV (assuming 10 copies per 22Rv1 genome [24]) and the results obtained from the TaqMan PCR are shown.

In the HIV patient screen, the quantity of leukocyte DNA per patient per PCR reaction averaged 362 ng (IQR 188–468 ng, 54–135×10^3^ genomes). We did not detect positive PCR signals from any of the patient DNA samples using either primer sets 1 or 2, indicating that neither XMRV nor any other X-MLV amplifiable with these primers were detectable in these samples. All samples were positive for GAPDH by TaqMan PCR at expected levels, showing that no PCR inhibitors were present.

## Discussion

In order to establish whether XMRV infects the human population and whether it is associated with human disease it is extremely important to be able to detect and quantitate XMRV specifically and sensitively. The majority of screens carried out so far have used highly sensitive nested-PCR protocols [1], [2], [4], [7], [8], [10], [11], [13]–[15], [17]. However, these sensitive nested protocols are prone to false-positives from contamination [14], [25] which can come from reagents, from X-MLVs growing as contaminants in human tumour cell lines, from amplicon contamination or from positive controls [26]–[28]. In order to minimise the risk of contamination in this study, real-time PCR was used instead of nested-PCR so that amplicons were not routinely exposed to the laboratory environment during the procedure. No 22Rv1 cells were grown in the laboratory until all screens were completed.

We could not detect XMRV or any other X-MLV sequences in HIV-1-positive patients using TaqMan PCR, suggesting that XMRV and related viruses are either entirely absent or at least extremely uncommon in this population cohort. There are several possible explanations for the discrepancy between this and some previous studies, including that XMRV does not in fact establish infection in human peripheral blood at detectable levels, or that the geographic differences between the cohort studied here and elsewhere are critical. Alternatively, the possibility that the positive findings reported by others were due to occult laboratory contamination should be seriously considered [29]. It has been shown that some anti-retroviral therapies can suppress XMRV infection [30], [31], but as approximately 20% of our cohort were untreated, this would mean that ∼108 patients were studied whose samples should have contained viral loads unsuppressed by any drugs that potentially interfere with XMRV (or X-MLV) replication. Our negative findings are entirely consistent with several other studies that have failed to detect any trace of XMRV infection in HIV-positive patients [14]–[17]. Henrich and colleagues [14] tested 43 HIV-infected patients (50% untreated) from Boston, Massachusetts using nested XMRV PCR with two different sets of primers and reported no positives. Barnes et al [15] failed to detect any XMRV in 230 HIV-1 patients from Switzerland and the United Kingdom using PCRs targeting XMRV *gag* or *env* sequences. 101 of the patients tested were not receiving antiretroviral drugs. Similarly, Kunstman et al [16], using real-time PCR, detected no XMRV sequences in the blood cells of 562 HIV-infected men enrolled in the Chicago component of the Multicenter AIDS Cohort Study. Finally, Cornelissen and colleagues [17] failed to detect any XMRV by nested PCR in 93 seminal plasma samples from 54 HIV-1-infected men living in the Netherlands. Other reported studies have focussed on detection of XMRV in patient groups that are different from those described here, for example, patients with chronic fatigue syndrome [2], [7]–[9] or prostate cancer [1], [6], [10], [11], or to assess whether XMRV could be responsible for conditions of unknown aetiology [12]–[14], [32].

At the current time there is no consensus on definitive strategies for testing for XMRV, or X-MLVs, and it remains unclear why the results of published studies differ so widely in their reported prevalence of XMRV in patient populations and healthy controls, although several independent studies have demonstrated how PCR contamination from mouse DNA or DNA from human cell lines infected with xenotropic MLVs might explain XMRV detection [26]–[29]. Other possibilities include differences in the patient groups studied, for example the cohort selection criteria as well as geographic factors. There are also differences in techniques used to detect the virus. Here, we used qPCR in order to rapidly screen a large patient group with a high degree of sensitivity. The use of primer set 2, which is not specific to XMRV and targets diverse X-MLV *gag* sequences, allowed us to detect viruses closely related to XMRV in addition to XMRV itself. By assaying DNA rather than RNA we sought proviral DNA rather than evidence for active replication. Nucleic acid testing remains the gold-standard for monitoring blood-borne viral diseases such as HIV and HCV, and is more specific than serological tests. Sequencing positive PCR products provides confirmatory evidence, and can illuminate when contamination has occurred [29].

In conclusion, this study failed to find any evidence of XMRV or X-MLV infection in a cohort of HIV-1-positive patients. In view of these negative findings in this highly susceptible group, we conclude that it is unlikely that XMRV or related viruses are circulating at a significant level, if at all, in HIV-1-positive patients in London or the healthy population.
